# Telephone reliability of the Frenchay Activity Index and EQ-5D amongst older adults

**DOI:** 10.1186/1477-7525-7-48

**Published:** 2009-05-29

**Authors:** Steven McPhail, Paul Lane, Trevor Russell, Sandra G Brauer, Steven Urry, Jan Jasiewicz, Peter Condie, Terry Haines

**Affiliations:** 1 Princess Alexandra Hospital, Ipswich Road, Woolloongabba, Queensland, Australia; 2 The University of Queensland, School of Health and Rehabilitation Sciences, St Lucia, Queensland, Australia; 3 Queensland University of Technology, Victoria Park Road, Kelvin Grove, Queensland, Australia; 4 Southern Health, Allied Health Research Unit, Kingston Centre, Cnr Warrigal and Kingston Roads, Cheltenham, Victoria, Australia; 5 Monash University, Physiotherapy Department, School of Primary Health Care, Monash University Peninsular Campus, Victoria, Australia

## Abstract

**Background:**

Older adults may find it problematic to attend hospital appointments due to the difficulty associated with travelling to, within and from a hospital facility for the purpose of a face-to-face assessment. This study aims to investigate equivalence between telephone and face-to-face administration for the Frenchay Activities Index (FAI) and the Euroqol-5D (EQ-5D) generic health-related quality of life instrument amongst an older adult population.

**Methods:**

Patients aged >65 (n = 53) who had been discharged to the community following an acute hospital admission underwent telephone administration of the FAI and EQ-5D instruments seven days prior to attending a hospital outpatient appointment where they completed a face-to-face administration of these instruments.

**Results:**

Overall, 40 subjects' datasets were complete for both assessments and included in analysis. The FAI items had high levels of agreement between the two modes of administration (item kappa's ranged 0.73 to 1.00) as did the EQ-5D (item kappa's ranged 0.67–0.83). For the FAI, EQ-5D VAS and EQ-5D utility score, intraclass correlation coefficients were 0.94, 0.58 and 0.82 respectively with paired t-tests indicating no significant systematic difference (p = 0.100, p = 0.690 and p = 0.290 respectively).

**Conclusion:**

Telephone administration of the FAI and EQ-5D instruments provides comparable results to face-to-face administration amongst older adults deemed to have cognitive functioning intact at a basic level, indicating that this is a suitable alternate approach for collection of this information.

## Background

Improving functional independence and health-related quality of life are two common and inter-related goals of health care services. These objectives are particularly important for services that cater for the needs of older adults. Evaluation of these services and ongoing monitoring of their patients requires that participation in functional activities and health-related quality of life be measured with an approach that is amenable to the clinical context. [1-3] A difficulty with evaluating these constructs is that many older adults find it difficult to attend hospitals or other health care settings for appointments. This is often due to the difficulty associated with travelling to, within and from a hospital facility for the purpose of a face-to-face assessment.[4,5] A viable alternative may be to complete relevant survey instruments via a telephone interview.

Different modes of administration of a range of self-reported outcomes have previously been investigated in a number of clinical populations. [6-15] Some of these investigations have found that equivalent responses were yielded from the different modes of administration [6-10], while others have found that responses were dependent on the mode of administration (such as self-completed postal surveys versus face-to-face administration) [11-15]. However, in order for telephone administration to be employed, it is important to demonstrate that equivalent answers would be elicited from respondents regardless of whether the mode of survey administration was face-to-face or via the telephone. This study aims to investigate equivalence between telephone and face-to-face administration of the Frenchay Activities Index[16] (FAI) and the Euroqol-5D[17] (EQ-5D) amongst sample of older adults who are accessing health care services. There have been no previous investigations of agreement between telephone and face-to-face administration of these instruments amongst older adults.

## Methods

### Design

Equivalence investigation between telephone and face-to-face administration for the FAI and the EQ-5D.

### Participants and setting

Older adults taking part in a larger evaluation of a novel home-based rehabilitation program following discharge from hospital participated in this study. Any person over the age of 65 years of age discharged from the geriatric rehabilitation, medical and surgical units of the Princess Alexandra Hospital during the study period were available for inclusion. Participants were excluded from the study if they had severe cardiac disease (unstable angina), cognitive impairment (Mini-Mental State Examination[18,19] score <23/30), restricted weight bearing status (non or partial weight bearing), aggressive behaviour, or referral for post-discharge community rehabilitation services.

### Measures

The FAI is a 15 item report of participation in functional activities recently undertaken by the respondent. Each of the 15 items require the participant to select one of four possible responses that best describes their recent level of participation in each nominated activity. Although the four possible responses varied between items, they generally ranged between 'never' and a more frequent response such as 'most days' or 'at least once weekly'. The longest time a respondent is required to recall is during item 11 which refers to the frequency of travel for the purpose of pleasure (for example a coach or rail trip) within the past 6 months. Each response was scored between zero (least frequent level of participation) and 3 (most frequent level of participation). An overall score out of 45 is calculated by summing each of the individual item scores. Evidence of sound validity and reliability have previously been reported for this instrument. [20-22]

The EQ-5D is a generic health-related quality of life measurement instrument consisting of 5 multiple choice questions, and a 100 point overall health state visual analogue scale (VAS). [17] The first 5 questions relate to mobility, personal care, usual activities, pain/discomfort and anxiety/depression respectively. The respondent is required to select one of three ordinal statements (e.g. no problems, some problems, unable) which best describes their health state in relation to these 5 domains. The responses from these 5 questions can be converted to a utility score where 0 and 1 represent death and perfect health respectively. To calculate the EQ-5D utility score the application of tariffs previously derived from population based surveys investigating preferences for all possible health state combinations is undertaken. The Dolan formula was used for utility calculation in this investigation[23]. Evidence of sound validity and reliability have previously been reported for this instrument. [17,24-30]

During telephone administration questions were read directly from the FAI and EQ-5D instruments. The first five EQ-5D questions were read verbatim as per telephone instructions [31], while the EQ-5D VAS component was modified slightly from the original version with a repetition of the question (for wording of EQ-5D VAS script see Additional file 1).

### Procedure

Hospital physiotherapists identified potential participants meeting the inclusion criteria who were about to be discharged from the hospital between March and December 2007 and sought verbal consent for the patient to be approached by a project research assistant. Pending this consent, the research assistant then provided a full description of the project to the patient and then sought written informed consent for participation. The research assistant then collected demographic information, including the FAI, EQ-5D and the Activity-specific Balance Confidence Scale[32] (a measure of how confident participants feel they can complete functional tasks without falling) prior to the patient being discharged from hospital.

Participants completed the FAI and EQ-5D via telephone interview with a research assistant (PL) seven days prior to an eight week post discharge outpatient review appointment at a tertiary hospital. The FAI and EQ-5D were then completed again at the subsequent outpatient appointment seven days later where the measures were administered face-to-face by the same research assistant. A seven day period was chosen so that participants would have a lower chance of remembering their response to the telephone-administered survey items when the time came for them to again complete the surveys via the face-to-face administration approach. A longer period was not chosen to minimise the risk that the participant's health would change in a measureable way during the between-assessment period.

This study was approved by the Human Research Ethics Committee of the Princess Alexandra Hospital, and the Medical Research Ethics Committee of The University of Queensland.

### Analysis

The Kappa statistic was used to describe the agreement between assessment approaches for individual items within the outcome measures examined. Confidence intervals for kappa statistics were calculated using bootstrap resampling (2000 replications of original sample size)[33,34]. The number of exact matches was also calculated for each item. For FAI and EQ-5D items the frequency of each possible response was computed to identify whether responses were distributed across the range of possible scores or whether they were at either end of the scales (potentially indicating ceiling or floor effects within an item).

FAI summative scores were calculated and EQ-5D response items were converted to utility scores using the Dolan formula.[23] Limits of agreement and intraclass correlation coefficients (ICC) were calculated to investigate agreement between assessment approaches for the FAI summary score, EQ-5D VAS and EQ-5D utility score. Paired t-tests were also employed to examine whether systematic differences between the two modes of administration existed for the FAI summary score, EQ-5D VAS and EQ-5D utility scores. Bland-Altman plots[35] are also presented for the FAI summary score, and EQ-5D VAS and utility scores.

## Results

Sixty-eight patients were screened by the recruiting research assistant after being referred by ward staff for potential participation in the study. All sixty eight met the inclusion criteria; however, fifteen did not provide informed consent. Fifty-three patients consented to participate. Forty subjects' datasets were complete for the FAI and EQ-5D at both assessments and were included in the analysis. Reasons for participants' incomplete sets of data excluded from analysis included unable to attend face-to-face reassessment on scheduled appointment day (8), not able to be contacted via telephone seven days prior to reassessment (3), readmitted to hospital with acute illness at time of assessment (1) and death (1). Participant demographics for patients included in analysis are displayed in Table 1. A high proportion of participants (90%) required a walking aid when outside their home and a substantial level of concern about falling over was present amongst this population.

**Table 1:** Participant demographics

Participants who complete both modes of administration	n = 40
Age – mean (sd)	79 (7.3)
Male	17 (42.5%)
Diagnosis category for recent hospital admission	
Orthopaedic	15 (37.5%)
Neurological	6 (15%)
Non-specific disabling impairment	12 (30%)
All other diagnoses combined	7 (17.5%)
English as first language	32 (80%)
Walking aid when outside home	
Nil	4 (10%)
Walking Stick/s	13 (32.5%)
4 Wheeled walking frame	21 (52.5%)
Other – Non-wheeled Walking aid	2 (5%)
The Activities-specific Balance Confidence Scale mean (sd) no confidence = 0 complete confidence = 100	54 (20)

Levels of agreement, and 95% confidence intervals, between telephone and face-to-face administration of the FAI and EQ-5D items are displayed in Table 2. For 11 out of 15 items of the FAI, kappa was >0.80 and was between 0.60 and 0.80 for the remaining 4 items. Similarly kappa for each of the EQ-5D domain items were either between 0.60 and 0.80 (3/5) or >0.80 (2/5). Mean scores, p-values from paired t-tests for differences in group means, limits of agreement and intraclass correlation coefficients between telephone and face-to-face administration for the FAI summary score, and for the EQ-5D VAS and utility scores are displayed in Table 3.

**Table 2:** Level of agreement (kappa) and frequency of exact matches between face-to-face and telephone administration of the Frenchay Activities Index and the EQ-5D items

	Level of agreement kappa (95% CI)	Frequency of exact matches (% out of 40)
Frenchay Activity Index items		
Prepare main meals	0.85 (0.68,0.97)	35 (88%)
Wash up	0.91 (0.82,0.98)	36 (90%)
Wash clothes	0.88 (0.70,0.98)	36 (90%)
Light housework	0.88 (0.72,1.00)	37 (93%)
Heavy housework	0.96 (0.87,1.00)	38 (95%)
Local shopping	0.89 (0.74,0.98)	36 (90%)
Social outing	0.73 (0.53,0.88)	30 (75%)
Walk outside >15 mins	0.73 (0.49,0.92)	33 (83%)
Active interest in hobby	0.78 (0.61,0.92)	33 (83%)
Drives/taxi/bus	1 (-,-)	40 (100%)
Outings/car ride	0.73 (0.39,0.93)	35 (88%)
Gardening	0.85 (0.73,0.96)	35 (88%)
Housework or car maintenance	0.92 (0.78,1.00)	38 (95%)
Reading books	0.83 (0.71,0.94)	32 (80%)
Gainful work	1 (-,-)	40 (100%)
EQ-5D items		
Mobility	0.67 (0.39,0.85)	33 (83%)
Personal care	0.87 (0.64,1.00)	38 (95%)
Usual activities	0.72 (0.48,0.90)	34 (85%)
Pain/discomfort	0.67 (0.45,0.86)	32 (80%)
Anxiety/depression	0.83 (0.64,0.96)	36 (90%)

**Table 3:** Intraclass correlation coefficient (ICC), mean scores, and limits of agreement (LOA) between telephone and face-to-face administration of the Frenchay Activity Index (FAI) and EQ-5D

				Limits of agreement			
					
Measure	ICC (95% CI)	telephone mean (95% CI)	face-to-face mean (95%CI)	Lower LOA (95% CI)	Mean difference (95% CI)	Upper LOA (95% CI)	p-value*
FAI – total	0.94 (0.89, 1.00)	18.7 (15.8, 21.6)	19.6 (16.8, 22.4)	-7.4 (-8.5, -6.4)	-0.9 (-1.9, 0.2)	5.7 (4.7, 6.7)	0.100
EQ-5D VAS	0.58 (0.23, 0.93)	67.6 (62.6, 72.7)	68.3 (63.0, 73.7)	-22.8 (-26.3, -19.3)	0.7 (-2.8, 4.2)	21.4 (17.9, 24.9)	0.690
EQ-5D utility	0.82 (0.65,0.98)	0.643 (0.559, 0.728)	0.619 (0.528, 0.709)	-0.268 (-0.314, -0.222)	-0.025 (-0.071, 0.022)	0.317 (0.271, 0.363)	0.290

Note: *a p-value < 0.05 indicates that a systematic difference exists (i.e. telephone responses were either consistently higher or consistently lower than face-to-face responses)

**Figure 1:**
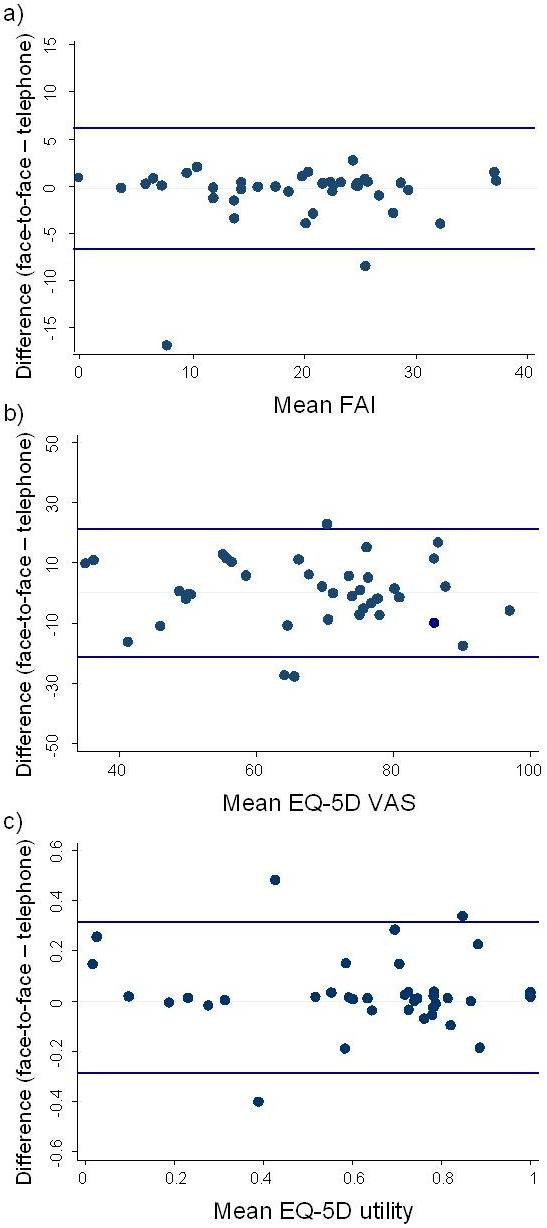
**Bland Altman plots for Frenchay Activity Index (a), EQ-5D VAS (b) and EQ-5D utility (c)**.

Two FAI items did not have participant answers distributed across the spectrum of responses. All (40) participants reported they did not undertake paid work while 33 reported that they never drive. The Bland-Altman plots for the FAI summary score, and EQ-5D VAS and utility scores are displayed (Figure 1) and do not indicate a relationship between overall score and level of agreement between telephone and face-to-face administration.

## Discussion

The FAI and EQ-5D generally had high levels of agreement between telephone and face-to-face administration of these instruments at both the individual item level and overall score. Within the EQ-5D instrument, the intraclass correlation coefficients and limits of agreement (Table 3) indicated agreement was lower for the VAS, than for the utility score. Greater variability within the VAS score is not surprising given its sensitivity to smaller amounts of change that might occur over a one week period relative to the discrete response items that combine to form the utility score.[36,37] While it is logical that some differences between modes of administration for the VAS may be, in part, attributable to the absence of visual representation of the scale during phone administration, agreement between responses from the two modes of administration for these instruments is comparable to test-retest reliability investigations for each the EQ-5D [24-27] and FAI [20-22] instruments where the same mode of administration was used at each assessment. This further strengthens the argument that telephone administration of these instruments is valid. In the same way, the mean differences, limits of agreement and paired t-tests indicate that although some variation existed between responses from each assessment for each instrument, no systematic difference was present. The high levels of agreement between the two modes of administration at the individual item level and overall scores indicate that telephone administration of these instruments is a valid method of obtaining this information from elderly patients in the community. The ability to collect this survey based information via the telephone offers viable and potentially more efficient and convenient approach to face-to-face assessment amongst older adults with cognition intact at a basic level.

The findings from this investigation are in line with previous reports of high levels of agreement between modes of administration for other related instruments [6-10]. Previous investigations which have not found high levels of agreement between modes of administration for survey instruments have often compared self-completed to interview administered modalities [11-15]. The high levels of agreement between the two modes of administration found in this investigation may be explained by similar nature of telephone and face-to-face administration of a survey instrument in comparison to self-completion.

A study of this nature will always have two key potential limitations that need to be counterbalanced. First is the risk that a participant may have anticipated the purpose of this study, recalled their original answer and responded in the same way when completing the questionnaires for the second time. Second is the risk that a participant's health may have measurably changed between the two assessment points. We believe that this study was more at risk of the second limitation than the first as we allowed a seven day washout period between assessments. This, combined with the shear number of items that a respondent would have had to remember correctly gave some protection against the memory-recall limitation. By doing so however, our results were likely to be more conservative than what could be expected in real life. Hence, given the nature of our design, we argue that the results of this investigation provide evidence that telephone administration of the FAI and EQ-5D (utility and VAS) instruments could be validly used in research or clinical practice.

The extrapolation of results from this investigation is limited somewhat as we focused our investigation solely upon older adults who are accessing health care services. Notably though, it is this population for whom telephone assessment of the constructs of participation in functional activities and health-related quality of life may be most important. We did however exclude participation by older adults with cognitive impairment as assessed by a Mini-Mental State Examination score of <23 out of 30. It is possible that older adults with cognitive impairment may not respond as consistently between the two administration approaches as our study sample did. Similarly some participants who were unable to attend the appointment to complete the face-to-face assessment may have had poorer health than those participants with complete datasets resulting in a slightly healthier sample. Along these lines, the study did not include many patients with poor to very poor self-rated health-related quality of life as a majority of responses were greater than 40 out of 100 on the VAS (Figure 1). It is possible that people with poor to very poor self-rated health may not respond as consistently between the two administration approaches as our study sample did. It is also noteworthy that some FAI items such as gainful work are likely to have low variance amongst older adult populations (as many are retired). Items like this may have exceptionally high levels of agreement, at least in part, due to a floor effect. Within this investigation this applied to both items with perfect agreement (gainful work and driving). However one could reason that the low variance observed in this investigation may often be present within these items amongst responses from this type of population. Thus the high level of agreement observed between modalities for these items may be reflective of what would occur in clinical, epidemiological and research settings that utilise these instruments amongst older adults.

Future investigations may consider the validity of telephone administration of other survey based instruments for the elderly as a way of reducing the burden of health assessments amongst this population. The ability to complete survey based instruments such as the FAI and EQ-5D via the telephone is likely to increase the feasibility of following up elderly patients in both clinical and research environments.

## Conclusion

This study has indicated that telephone and face-to-face administration of the Frenchay Activity Index and EQ-5D yields comparable responses amongst older adults with cognition intact at a basic level.

## Competing interests

The authors declare that they have no competing interests.

## Authors' contributions

All authors contributed to the conception of research idea and planning of process. PL contributed to data collection. SM contributed to data analysis. SM and TH were involved in manuscript preparation. All authors contributed to manuscript review, appraisal and editing.

## Supplementary Material

Additional file 1 **Verbal description for EQ-5D VAS (italicized text indicates wording has been added to or adapted from the original EQ-5D text to facilitate phone administration).** Click here for file

## References

[B1] Kind P (2001). Measuring quality of life in evaluating clinical interventions: an overview. Ann Med.

[B2] Hickey A, Barker M, McGee H, O'Boyle C (2005). Measuring health-related quality of life in older patient populations: a review of current approaches. Pharmacoeconomics.

[B3] McPhail S, Beller E, Haines T (2008). Two perspectives of proxy reporting of health-related quality of life using the Euroqol-5D, an investigation of agreement. Med Care.

[B4] Forrest CB, Starfield B (1998). Entry into primary care and continuity: the effects of access. American journal of public health.

[B5] Allsup SJ, Gosney MA (2002). Difficulties of recruitment for a randomized controlled trial involving influenza vaccination in healthy older people. Gerontology.

[B6] Pinto-Meza A, Serrano-Blanco A, Penarrubia MT, Blanco E, Haro JM (2005). Assessing depression in primary care with the PHQ-9: can it be carried out over the telephone?. J Gen Intern Med.

[B7] Barker RN, Amsters DI, Kendall MD, Pershouse KJ, Haines TP (2007). Reliability of the clinical outcome variables scale when administered via telephone to assess mobility in people with spinal cord injury. Archives of physical medicine and rehabilitation.

[B8] Maisto SA, Conigliaro JC, Gordon AJ, McGinnis KA, Justice AC (2008). An experimental study of the agreement of self-administration and telephone administration of the Timeline Followback interview. J Stud Alcohol Drugs.

[B9] Aziz MA, Kenford S (2004). Comparability of telephone and face-to-face interviews in assessing patients with posttraumatic stress disorder. Journal of psychiatric practice.

[B10] Verghese J, Katz MJ, Derby CA, Kuslansky G, Hall CB, Lipton RB (2004). Reliability and validity of a telephone-based mobility assessment questionnaire. Age Ageing.

[B11] McHorney CA, Kosinski M, Ware JE (1994). Comparisons of the costs and quality of norms for the SF-36 health survey collected by mail versus telephone interview: results from a national survey. Med Care.

[B12] Bowling A (2005). Mode of questionnaire administration can have serious effects on data quality. Journal of public health (Oxford, England).

[B13] Grootendorst PV, Feeny DH, Furlong W (1997). Does it matter whom and how you ask? inter- and intra-rater agreement in the Ontario Health Survey. J Clin Epidemiol.

[B14] Bowling A, Bond M, Jenkinson C, Lamping DL (1999). Short Form 36 (SF-36) Health Survey questionnaire: which normative data should be used? Comparisons between the norms provided by the Omnibus Survey in Britain, the Health Survey for England and the Oxford Healthy Life Survey. Journal of public health medicine.

[B15] Hanmer J, Hays RD, Fryback DG (2007). Mode of administration is important in US national estimates of health-related quality of life. Med Care.

[B16] Holbrook M, Skilbeck CE (1983). An activities index for use with stroke patients. Age and ageing.

[B17] Rabin R, de Charro F (2001). EQ-5D: a measure of health status from the EuroQol Group. Ann Med.

[B18] Folstein M, Folstein S, McHugh P (1975). Mini-Mental State: a practical method for grading the cognitive state of patients for the clinician. Journal of Psychiatric Research.

[B19] Tombaugh T, McIntyre N (1992). The mini-mental state examination: a comprehensive review. Journal of the American Geriatrics Society.

[B20] Turnbull JC, Kersten P, Habib M, McLellan L, Mullee MA, George S (2000). Validation of the Frenchay Activities Index in a general population aged 16 years and older. Archives of physical medicine and rehabilitation.

[B21] Green J, Forster A, Young J (2001). A test-retest reliability study of the Barthel Index, the Rivermead Mobility Index, the Nottingham Extended Activities of Daily Living Scale and the Frenchay Activities Index in stroke patients. Disability and rehabilitation.

[B22] Piercy M, Carter J, Mant J, Wade DT (2000). Inter-rater reliability of the Frenchay activities index in patients with stroke and their careers. Clinical rehabilitation.

[B23] Dolan P, Roberts J (2002). Modelling valuations for Eq-5d health states: an alternative model using differences in valuations. Med Care.

[B24] Unal G, de Boer JB, Borsboom GJ, Brouwer JT, Essink-Bot M, de Man RA (2001). A psychometric comparison of health-related quality of life measures in chronic liver disease. Journal of clinical epidemiology.

[B25] Fransen M, Edmonds J (1999). Reliability and validity of the EuroQol in patients with osteoarthritis of the knee. Rheumatology (Oxford, England).

[B26] Dorman P, Slattery J, Farrell B, Dennis M, Sandercock P (1998). Qualitative comparison of the reliability of health status assessments with the EuroQol and SF-36 questionnaires after stroke. United Kingdom Collaborators in the International Stroke Trial. Stroke; a journal of cerebral circulation.

[B27] Hurst NP, Kind P, Ruta D, Hunter M, Stubbings A (1997). Measuring health-related quality of life in rheumatoid arthritis: validity, responsiveness and reliability of EuroQol (EQ-5D). British journal of rheumatology.

[B28] Coons SJ, Rao S, Keininger DL, Hays RD (2000). A comparative review of generic quality-of-life instruments. PharmacoEconomics.

[B29] Konig HH, Ulshofer A, Gregor M, von Tirpitz C, Reinshagen M, Adler G, Leidl R (2002). Validation of the EuroQol questionnaire in patients with inflammatory bowel disease. European journal of gastroenterology & hepatology.

[B30] Schweikert B, Hahmann H, Leidl R (2006). Validation of the EuroQol questionnaire in cardiac rehabilitation. Heart (British Cardiac Society).

[B31] Brooks R, Brooks R, Rabin R, De Charro F (2003). The measurement and valuation of health status using EQ-5D: A European perspective. Evidence from the Euro-Qol BIO MED Research Programme.

[B32] Powell LE, Myers AM (1995). The Activities-specific Balance Confidence (ABC) Scale. The journals of gerontology.

[B33] Efron B, Tibshirani R (1993). An introduction to the bootstrap.

[B34] Lee J, Fung KP (1993). Confidence interval of the kappa coefficient by bootstrap resampling. Psychiatry Res.

[B35] Bland JM, Altman DG (1986). Statistical methods for assessing agreement between two methods of clinical measurement. Lancet.

[B36] Krabbe PF, Peerenboom L, Langenhoff BS, Ruers TJ (2004). Responsiveness of the generic EQ-5D summary measure compared to the disease-specific EORTC QLQ C-30. Qual Life Res.

[B37] Wu AW, Jacobson KL, Frick KD, Clark R, Revicki DA, Freedberg KA, Scott-Lennox J, Feinberg J (2002). Validity and responsiveness of the euroqol as a measure of health-related quality of life in people enrolled in an AIDS clinical trial. Qual Life Res.

