# Supplementary material for: Telephone reliability of the Frenchay Activity Index and EQ-5D amongst older adults
Source: Health Qual Life Outcomes. 2009 May 29;7:48. doi: 10.1186/1477-7525-7-48 (PMC2695435; doi:10.1186/1477-7525-7-48)
Supplement: Additional file 1 — Verbal description for EQ-5D VAS (italicized text indicates wording has been added to or adapted from the original EQ-5D text to facilitate phone administration). [file 1477-7525-7-48-S1.doc]

# Additional file 1

**Verbal description for EQ-5D VAS** *(italicized text indicates wording has been added to or adapted from the original EQ-5D text to facilitate phone administration).*

To help you say how good or bad your health state is, *I have a scale in front of me,* (rather like a thermometer) on which the best health state you can imagine is marked 100 and the worst health state you can imagine is marked 0.

We would like you to indicate on this scale how good or bad your own health is today, in your opinion. Please do this by *telling me where I should draw a mark* on this scale between 0 and 100 to indicate how good or bad your health state is today. *Remember the best health state you can imagine is marked 100 and the worst health state you can imagine is marked 0. In your opinion, which number on this scale from 0 to 100 should I mark your own health state as, today.*
